# Effect of various bacteriological media on the plaque morphology of *Staphylococcus* and *Vibrio* phages

**DOI:** 10.1099/acmi.0.000036

**Published:** 2019-06-17

**Authors:** Nachimuthu Ramesh, Loganathan Archana, Madhav Madurantakam Royam, Prasanth Manohar, Kandasamy Eniyan

**Affiliations:** ^1^ Antibiotic Resistance and Phage Therapy Laboratory, School of Bio Science and Technology, Vellore Institute of Technology, Vellore, Tamil Nadu, India

**Keywords:** bacteriophage, plaque assay, plaque morphology, phage therapy, lytic-lysogenic shift

## Abstract

The influence of media composition on the life cycle of bacteriophages to exhibit diverse plaque morphology on various bacteriological media was investigated by a double agar overlay method. Both *Staphylococcus aureus* phage and *Vibrio parahaemolyticus* phage showed altered plaque morphology from small to large and from clear to turbid, in different culture media used for the double agar overlay method.

## Introduction

Phage therapy is re-emerging as one of the effective alternative therapies against multi-drug-resistant bacterial infections. Studies on bacteriophages are gaining renewed interest because phages are believed to be an effective therapeutic agent, and phage-based products have been receiving approval in recent years [1]. Usually, the phage-enrichment method is used for phage isolation against the desired bacterial host. Even if a single phage, which is capable of infecting the bacterial host, is present in the sample, it can result in phage multiplication and produce phage progeny, which can be assessed using plating techniques. Further, the presence of phages are quantitatively studied by the double agar overlay method [2, 3]. Double agar overlay is a plaque assay technique, which is performed to determine the number of phages present and to study the morphology of plaques formed due to the activity of the phages against the host bacteria. In the double overlay agar method, host bacteria are mixed with the phages in a low concentration agar (soft agar), which is then overlayed on to a hard agar surface in a petri plate.

Plaque morphologies observed during the double agar overlay were used for differentiating the lytic and lysogenic phages because it is believed that lytic phages have a tendency to produce clear plaques and lysogenic phages produce turbid or bulls-eyed plaques [4, 5]. Although a plaque-based assay is commonly used for identification of a bacteriophage, there is a lack of understanding about the morphologies of plaque formed when different compositions of bacteriological medium are used. Therefore, this study aims to differentiate the plaque morphologies of two different bacteriophages formed on various bacteriological media.

## Methods

A phage-enrichment method was used to isolate bacteriophages against the host bacterial strains. The *Staphylococcus aureus* isolate used in this study was isolated from a clinical sample collected from a diagnostic centre in Chennai. For isolation of the phage against *S. aureus *, 10 ml of the water sample was added to 5 ml of the *S. aureus* bacterial culture and incubated for 20–24 h at 37 °C to enrich the phages capable of infecting the bacteria.

*Vibrio parahaemolyticus* was isolated from a water sample collected from an aqua-culture pond in Vellore. Similarly, for isolation of the phage against *V. parahaemolyticus* **,** 10 ml of the water sample was added to 5 ml of the *V. parahaemolyticus* bacterial culture and incubated for 20–24 h at 37 °C to enrich the phages capable of infecting the bacteria. The presence of phage was confirmed by spot test and double agar overlay method.

To understand the effect of various bacteriological media on the plaque morphology, we conducted the analysis using various bacteriological media (HiMedia, India) such as nutrient agar (NA), Luria–Bertani agar (LB), brain heart infusion agar (BHI), Mueller–Hinton agar (MHA), tryptic soy agar (TSA) and all the media were tested with and without the addition of CaCl_2_ for both the *S. aureus* phage (SAP1) and the *V. parahaemolyticus* phage (VPP1). Briefly, the phage–bacteria mixture (2 : 1) was added to the soft agar (0.75 %) and plated over the pre-prepared hard agar (1.5 %) plate and the plaque morphologies were determined after incubating the plates for 16–20 h at 37 °C. Experiments were performed in triplicates and statistical analyses were performed using the mean (sd).

## Results

**Fig. 1.**
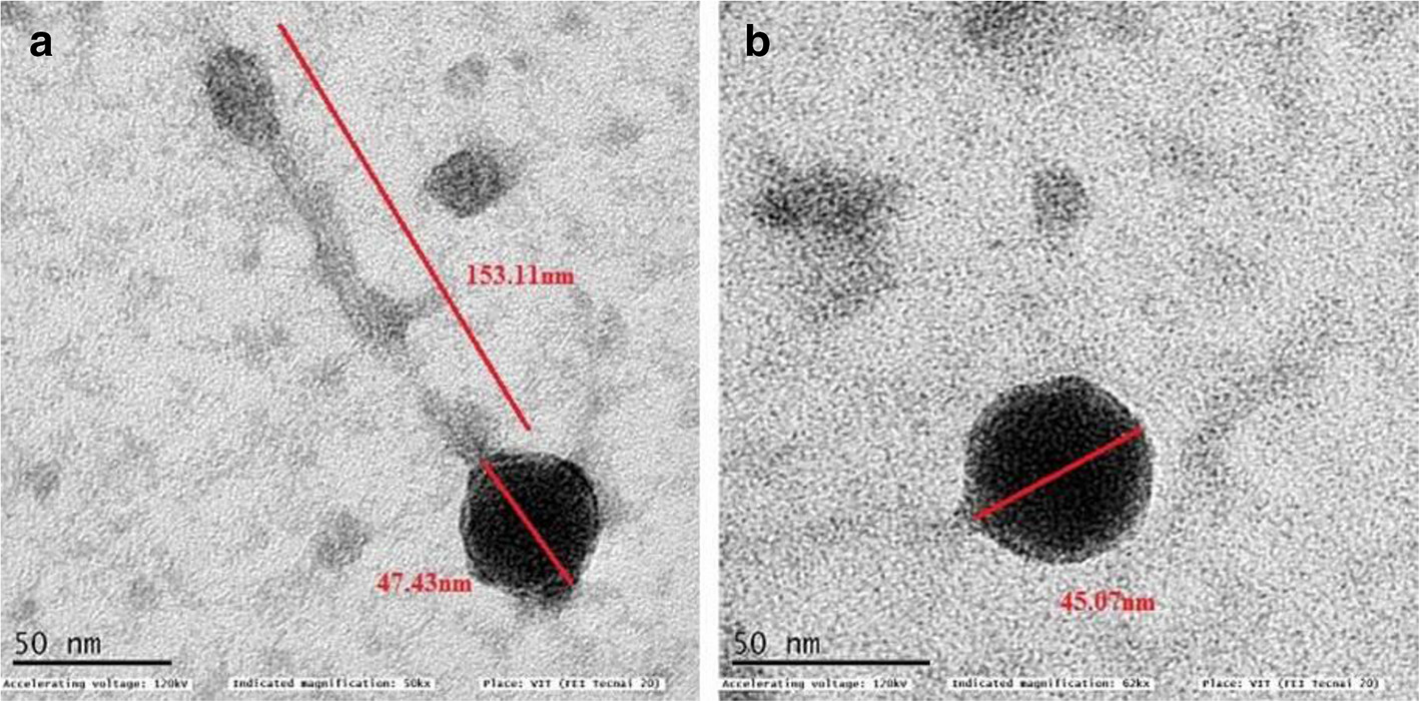
Transmission electron micrographs of (a) SAP1 and (b) VPP1. Scale bar, 50 nm.

**Fig. 2.**
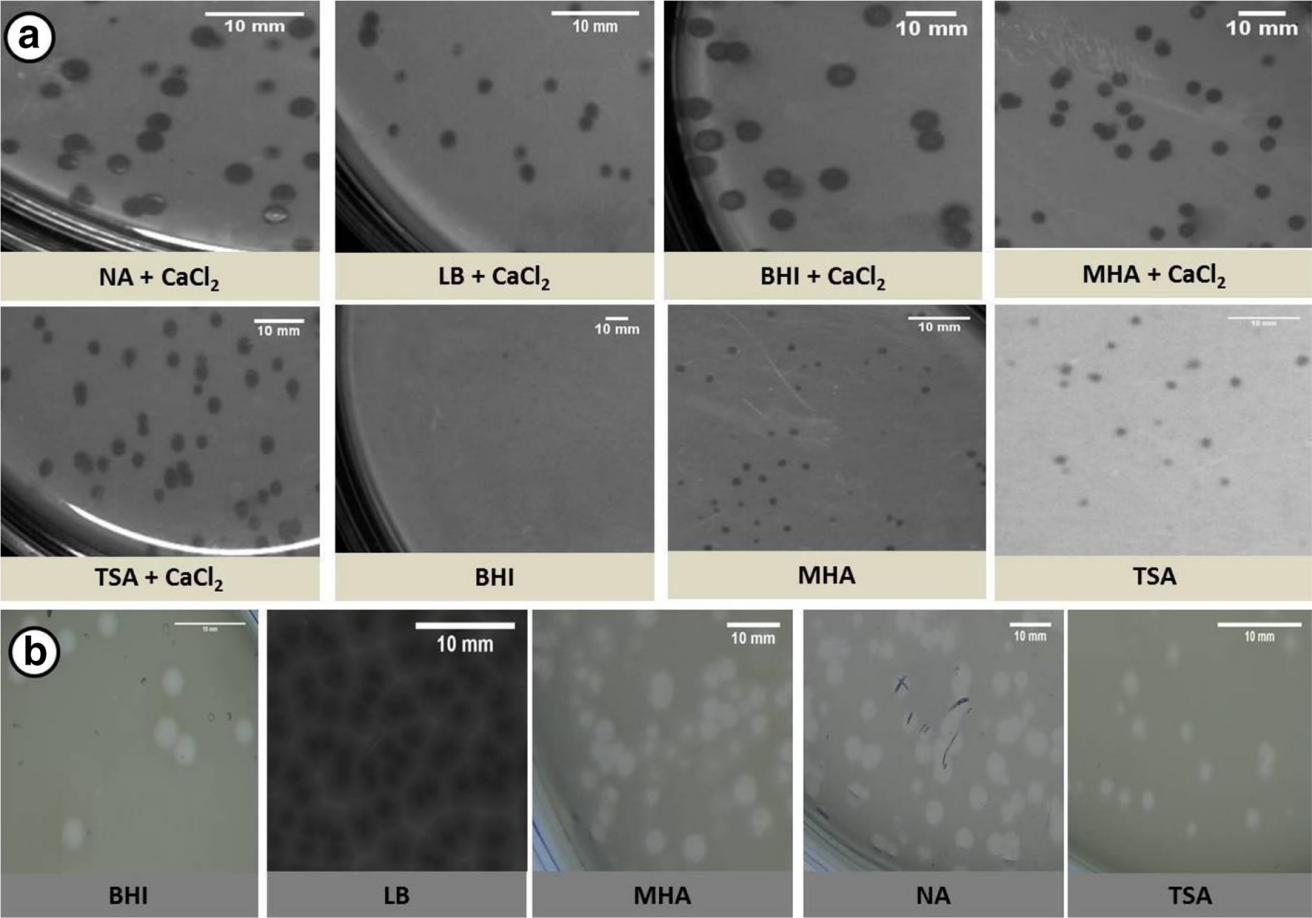
Plaque morphologies of (a) SAP1 and (b) VPP1 as observed on different bacteriological medium. Nutrient agar (NA), Luria–Bertani agar (LB), brain heart infusion agar (BHI), Mueller–Hinton agar (MHA), tryptic soy agar (TSA), CaCl_2_ (calcium chloride). Scale bar, 10 mm.

SAP1 was isolated from lake water, and the isolated phage was confirmed for their antibacterial activity using the spot test and plaque assay. Transmission electron microscopy (TEM) analysis showed that SAP1 belongs to the *Siphoviridae* family (Fig. 1). The double agar overlay method was performed for the isolated phage, SAP1, to determine plaque morphology on various bacteriological media. Accordingly, the plaques formed on different media showed vivid morphology, and they were classified from very small to large and clear to completely turbid plaques (Table 1). The most observable characteristic of the SAP1 phage was that they did not produce plaques on NA and LB without the supplementation of CaCl_2_, whereas they produced a very small plaque of 1±0.2, 1±0.3, 1±0.2 mm in size on TSA, MHA and BHI media, respectively, without CaCl_2_ supplementation. In CaCl_2 _supplemented media such as in LB agar it produced a very clear, lytic and small circular plaque of 1.5±0.3 mm in size; MHA, NA and TSA showed medium plaques of 2.5±0.4, 2.5±0.3, 2.5±0.3 mm in size with a turbid halo, while BHI showed a larger plaque of 5.5±0.6 mm with a turbid halo (Fig. 2). Another phage, VPP1, was isolated from sewage samples and TEM analysis showed VPP1 belongs to the *Podoviridae* family (Fig. 1). VPP1 produced plaques even in the absence of CaCl_2_ showed a plaque morphology from medium to large, bulls-eyed and regular plaque of 1.5–3.5 mm in size, with large, circular and turbid plaques with 3.5±0.4 and 3.5±0.5 mm size in NA and BHI, respectively; bulls-eyed plaques with 3.5±0.2 mm size in LB; medium, hazy and circular plaques with 1.5±0.2 mm size in TSA; large, hazy and turbid plaques with 3.5±0.4 mm size in MHA (Fig. 2). Interestingly, the absence of CaCl_2_ did not have any effect on the plaques formed by VPP1 phage.

**Table 1.** Characteristics of plaques as observed on different bacteriological medium

S. No.	Medium	SAP1 (Mean±sd)	VPP1 (Mean±sd)
1.	**na**	No plaques	3.5±0.4 mm turbid plaques
2.	**NA- CaCl_2_**	2.5±0.3 mm turbid plaques	3.5±0.3 mm turbid plaques
3.	**LB**	No plaques	3.5±0.2 mm bulls-eyed plaques
4.	**LB- CaCl_2_**	1.5±0.3 mm clear plaques	3.5±0.3 mm turbid plaques
5.	**BHI**	1±0.2 mm clear plaques	3.5±0.5 mm turbid plaques
6.	**BHI-CaCl_2_**	5.5±0.6 mm turbid plaques	3.5±0.4 mm turbid plaques
7.	**MHA**	1±0.3 mm plaques	3.5±0.4 mm turbid plaques
8.	**MHA-CaCl_2_**	2.5±0.4 mm turbid plaques	3.5±0.5 mm turbid plaques
9.	**TSA**	1±0.2 mm clear plaques	1.5±0.2 mm turbid plaques
10.	**TSA-CaCl_2_**	2.5±0.3 mm turbid plaques	1.5±0.3 mm turbid plaques

Highlighting represents the difference in plaque morphology observed when CaCl_2_ is supplemented.

BHI, Brain Heart Infusion agar; CaCl_2_, calcium chloride; LB, Luria Bertani agar; MHA, Muller Hinton agar; NA, Nutrient agar; SAP1, *Staphylococcus aureus* phage; TSA, Tryptic Soy agar; VPP1, *Vibrio parahaemolyticus* phage.

## Discussion

From the above study, we conclude that the composition of the bacteriological media has a significant effect on the plaque morphology, showing plaques of clear to turbid morphologies with varying size. Some supplements such as CaCl_2_ was also found to cause substantial differences in plaque morphologies observed for the *S. aureus* phage while for the *V. parahaemolyticus* phage, CaCl_2_ supplement is not important [6]. LB showed very small and clear plaques in both the phages that failed to show the turbid haloes. On the other hand, BHI, MHA and TSA showed a large and turbid halo. This variation in the plaque morphology has occurred as a result of nutrient variations in the media. Possible reasons could be: (i) almost all the studies on bacteriophages have been performed under favourable conditions for the bacteria but there is a possibility that these conditions may not be optimal for the bacteriophage multiplication, (ii) as reported earlier, the level of glucose production by host bacterium in BHI, TSA and MHA is high enough to influence the bacterial growth eventually leading to intracellular cAMP causing increased phage production [7]. As explained previously,the growth media plays a vital role in controlling the phage production, which coincides with our study showing that nutrients in the growth medium is a major factor in deciding the plaque morphology [8]. The requirement of the micronutrient, macronutrient and divalent cations remains specific for each phage. Thus, the double agar overlay method becomes a preliminary assay for determining the phage life cycle (based on plaque morphology). Although there are high-end technologies, such as whole-genome sequencing, meta-genome sequencing and other techniques available for determining the phage life cycle, these techniques appear to be highly expensive for smaller laboratories (minimal fund) where double agar overlay is still conformational evidence [9, 10]. The determination of the phage life cycle seems to be very important for the phages that are used for the therapeutic purpose. Hence, there is a need for complete assessment for the proper validation of the phage life cycle, which cannot be entirely reliable on the double agar overlay method. Further studies are required to confirm that media composition and other environmental factors can influence the plaque morphology formed during the double agar overlay method. 
